# Association of TIMP4 gene variants with steroid-induced osteonecrosis of the femoral head in the population of northern China

**DOI:** 10.7717/peerj.6270

**Published:** 2019-01-24

**Authors:** Jiaqi Wang, Feimeng An, Yuju Cao, Hongyan Gao, Mingqi Sun, Chao Ma, Hao Wu, Baoxin Zhang, Wanlin Liu, Jianzhong Wang

**Affiliations:** 1 Inner Mongolia Medical University, Hohhot, China; 2 Zhengzhou Traditional Chinese Medicine Traumatology Hospital, Zhengzhou, China; 3 The Second Affiliated Hospital of Inner Mongolia Medical University, Hohhot, China

**Keywords:** Tissue inhibitors of metalloprotease-4, Osteonecrosis of the femoral head, Single-nucleotide polymorphisms, Gene variants, Clinical phenotypes, Association study

## Abstract

**Background:**

In clinical treatment, the use of steroid hormones is an important etiological factor of non-traumatic osteonecrosis of the femoral head (ONFH) risk. As an endogenous inhibitor of matrix metalloproteinases (MMPs) in the extracellular matrix, the expression of tissue inhibitors of metalloprotease-4 (TIMP4) plays an essential role in cartilage and bone tissue damage and remodeling, vasculitis formation, intravascular thrombosis, and lipid metabolism.

**Methods:**

This study aimed to detect the association between TIMP4 polymorphism and steroid-induced ONFH. We genotyped seven single-nucleotide polymorphisms (SNPs) in TIMP4 genes and analyzed the association with steroid-induced ONFH from 286 steroid-induced ONFH patients and 309 normal individuals.

**Results:**

We performed allelic model analysis and found that the minor alleles of five SNPs (rs99365, rs308952, rs3817004, rs2279750, and rs3755724) were associated with decreased steroid-induced ONFH (*p* = 0.02, *p* = 0.03, *p* = 0.04, *p* = 0.01, *p* = 0.04, respectively). rs2279750 showed a significant association with decreased risk of steroid-induced ONFH in the Dominant and Log-additive models (*p* = 0.042, *p* = 0.028, respectively), and rs9935, rs30892, and rs3817004 were associated with decreased risk in the Log-additive model (*p* = 0.038, *p* = 0.044, *p* = 0.042, respectively). In further stratification analysis, TIMP4 gene variants showed a significant association with steroid-induced ONFH in gender under the genotypes. Haplotype analysis also revealed that “TCAGAC” and “CCGGAA” sequences have protective effect on steroid-induced ONFH.

**Conclusion:**

Our results indicate that five TIMP4 SNPs (rs99365, rs308952, rs3817004 rs2279750, and rs3755724) are significantly associated with decreased risk of steroid-induced ONFH in the population of northern China.

## Introduction

Osteonecrosis of the femoral head (ONFH) is a common disease in bone and joint surgery, and is usually clinically classified as traumatic ONFH and atraumatic ONFH. The occurrence of ONFH is closely associated with several factors, including transcervical fracture, hormone intake, chronic alcoholism, sickle cell disease, and decompression sickness (Shibahara et al., 2000). From the initial hip osteoarthritis, followed by the eventual collapse and deformation of the femoral head, and then the eventual final hip dysfunction, ONFH has a high disability rate if not treated early. There are many effective therapies for ONFH, such as drug intervention and hip replacement therapy, and the number of patients with ONFH who seek treatment is gradually increasing. In 2004, a Japanese survey reported that nearly 11,400 ONFH patients require treatment yearly, with about 2,200 new cases reported each year (Kubo et al., 2016). In China, there are about 7 million patients with ONFH, and more than 100,000–200,000 new cases annually (Yin et al., 2014). In 1953, Pietrogrande and Marino first reported association of the use of glucocorticoids with ONFH (Pietrogrande & Marino, 1953). The treatment of some diseases such as asthma, rheumatoid arthritis, and lymphoid malignancies are dependent on the use of steroid hormones, and steroid intake was identified as the important cause of ONFH.

The development and progression of steroid-induced ONFH is a complex dynamic process mediated by multiple factors and signaling pathways, and is regulated by different genes. The disruption of the blood supply of the femoral head causes bone cell death, bone marrow edema, and eventually bone destruction and the collapse of the femoral head. Possible mechanisms that stimulate this process include high blood coagulation, thrombosis in blood vessels, and arterial contraction of the blood supply of the femoral head (Janke et al., 2013). Jaffe et al. first described the effects of corticosteroids to increase fat deposition in the intramedullary tissue of the femoral head, which can induce hyperlipidemia, resulting in increased cortical pressure, blood flow restriction, and local ischemia (Jaffe et al., 1972). With decreased number and activity of osteoblasts, apoptosis may also contribute to the pathological process of ONFH (Zhu et al., 2011). In recent years, single-nucleotide polymorphisms of some genes have been linked to steroid-induced ONFH. Research has been confirmed that variants of endothelial NO synthase (eNOS), Sex determining region Y-box 9 (SOX9),paraoxonase-1 (PON-1), Osteoprotegerin (OPG), and receptor activator of nuclear factor-kappa B ligand (RANKL) genes were closely correlated with the development of ONFH (Song & Du, 2016; Gagala et al., 2013; Wang et al., 2013; Li et al., 2016).

Matrix metall oproteinases (MMPs) and tissue inhibitors of metalloproteases (TIMPs) family are   widely   present in the extracellular matrix (ECM) of all tissues of our body, and also participate in the pathological processes of diseases. TIMPs can regulate proteolysis of the extracellular matrix by inhibiting MMP-dependent ECM proteolysis (Arpino, Brock & Gill, 2015). The extracellular matrix is an important extracellular component that provides structural and biological support for the surrounding cells, and its components are involved in cell adhesion, cell spreading, and cortical actin cytoskeleton organization (Gao et al., 2014). Guo et al. (2004) confirmed that the over expression of TIMP4 inhibited the formation of neointima of the carotid artery, inhibited the migration of vascular smooth muscle cells, and induced apoptosis. TIMP4 can contribute to hemostasis of the extracellular matrix in a tissue-specific manner (Gomez et al., 1997). The balance between MMP and TIMP plays an important role in regulating the balance of cartilage production and degradation. Expression of TIMP1 and TIMP4 was decreased significantly in osteoarthritis patients, which resulted in increased metalloproteinase, leading to pathological cartilage destruction (Kevorkian et al., 2004). Grässel, et al. confirmed that gene expression rates of MMP-2 and TIMP-1 were higher in bone tissue of ONFH, and the increase of MMP-2 biosynthesis and activation may counter-acting the production of new bone matrix (Grassel et al., 2010). In a study of a population of northern China, Jieli, Du. et al. indicated that MMP8 polymorphisms are significantly associated with susceptibility to steroid-induced ONFH (Du et al., 2016).

In the pathogenesis of steroid-induced ONFH, different signaling pathways and transcription factors play a key role. However, it is not clear whether mutation of the TIMP4 gene is involved in the development of steroid-induced ONFH. In this work, we analyzed SNP alleles, genotypes, and haplotypes in 309 patients and 286 controls to explore the potential association between TIMP4 single-nucleotide polymorphisms and steroid-induced ONFH in the population of northern China.

## Materials and Methods

### Study participants

A total of 595 people (309 steroid-induced ONFH patients and 286 normal individuals) were recruited from the Zhengzhou Traditional Chinese Medicine Traumatology Hospital between September 2014 and January 2016. All participants were Han Chinese from northern China living in or around Zhengzhou and were randomly selected without age or sex restriction.

Steroid-induced ONFH patients who participated in the research not only had common clinical manifestations of hip pain, lower limb muscle atrophy, claudication, and joint dysfunction , but also have received long-term steroid intake of more than 16 mg per day or high-dose steroid impulsion treatments for more than 1 week before the appearance of these symptoms (Du et al., 2016). Each patient was subjected to X-ray examination and additional magnetic resonance imaging (MRI) analysis if necessary. Diagnostic criteria for imaging include increased subchondral bone density, normal structural changes of the trabecular bone, hip joint narrowness, and the collapse of the femoral head. Similarly, we applied set strict exclusion criteria as follows: patients who did not strictly conform to the diagnostic criteria for steroid-induced ONFH, patients of traumatic ONFH and other hip diseases; patients with an alcohol intake of more than 400 ml per week (Du et al., 2016); and patients with a significant history of disease or severe chronic diseases such as renal insufficiency, diabetes mellitus, cardiovascular and cerebrovascular diseases. Health control subjects were defined in the following criteria: they had no symptoms of hip disease and no history of thromboembolic disease. Individuals with severe chronic diseases such as renal insufficiency, diabetes mellitus, cardiovascular and cerebrovascular diseases, and significant familial hereditary disease will be excluded. We collected the epidemiological information by using a standardized questionnaire and collected clinical information from medical records and Imaging examination.

The case-control study was conducted in strict accordance with the principles of the Declaration Helsinki and was approved by the Ethics Committee of the Inner Mongolia Medical University. Written informed consent was obtained from all participants before their participation in the study. All 595 study participants signed informed consent forms related to this study.

### SNP selection and genotyping

Seven SNPs were selected from TIMP4 genes, with allelic frequencies >5% in the HapMap Chinese Han Beijing (CHB) population. The majority of the seven SNPs had not been previously reported. However, rs3755724 was related to some other disease, such as Kawasaki disease (Ban et al., 2009), hepatocellular carcinoma (Tsai et al., 2014), and focal epilepsy (Haerian et al., 2015). Five milliliters peripheral blood samples were collected in ethylenediamine-tetra-acetic acid (EDTA) containing tubes, centrifuged and stored at −80 °C. The GoldMag-Mini Whole Blood Genomic DNA Purification Kit (GoldMag Ltd., Xi’an, China) was used for genomic DNA   extraction from whole blood samples according to the manufacturer’s protocol. The concentration of DNA was measured by spectrometry (DU530UV/VIS spectrophotometer; Beckman Instruments, Fullerton, CA, USA). Sequenom MassARRAY Assay Design 3.0 Software was selected to design a Multiplexed SNP MassEXTEND assay. The Sequenom MassARRAY platform was used to perform the SNP genotyping according to the manufacturer’s protocol.

### Statistical analysis

The genotype frequency distribution of all seven SNPs conformed to Hardy-Weinberg equilibrium in the control subjects according to the exact test. A value of *P* ≤ 0.05 was considered statistically significant. We compared the allelic and genotypic frequencies of steroid-induced ONFH patients and controls using Chi-squared test/Fisher’s exact tests. In haplotype model analysis, PLINK software was used to assess the association between SNPs in TIMP4 and the risk of steroid-induced ONFH under five models (codominant, dominant, recessive, and Log-additive genetic models). Odds ratios (ORs) and 95% confidence intervals (CIs) were calculated by unconditional logistic regression analysis with adjustment for gender and age. Finally, linkage disequilibrium, haplotype construction, and genetic associations at polymorphism loci were estimated by using the Haploview software package (version 4.2) and the SHEsis software platform (http://www.nhgg.org/analysis/).

## Results

### Demographic characteristics of the study population

A total of 595 people participated in this case-control study of 286 patients (113 female, 173 male) with steroid-induced ONFH and 309 healthy controls (112 female, 197 male). The more detailed basic characteristics of the study participants are listed in Table 1. The age distribution factor differed between the case and control groups, so the variable was adjusted in the subsequent multivariate unconditional logistic regression analysis to eliminate the residual confounding effects.

**Table 1:** Characteristics of cases and controls in this study. The basic characteristics of the study participants are listed in Table 1.

**Variable(s)**	**Case** (*n* = 286)	**Control** (*n* = 309)	*p***value**
**Sex N (%)**			0.412^a^
Male	173(46.8%)	197(53.2%)	
Female	113(50.2%)	112(49.8%)	
**Age, year(mean ± SD)**	41.83 ±13.115	48.75 ±8.422	<0.001^b^
**Clinical stages**			
Stage II	64(22.4%)		
Stage III	126(44.1%)		
Stage IV	96(33.5%)		
**Hip lesions**			
Unilateral	83(29.1%)		
Bilateral	203(70.9%)		

**Notes.**

a Two-sided Chi-squared test.

b Independent samples *t* test.

### Risk assessment between TIMP4 Allele frequencies and steroid-induced ONFH

Seven SNPs in the TIMP4 gene (rs99365, rs17035945, rs308952, rs3817004, rs28897670, rs2279750, and rs3755724) were selected for experimental studies.  The statistics of the allelic distributions, minor allele frequency (MAF), odds ratios (ORs), 95% confdence intervals (95% CIs) and the *P-* values of alleles are presented in Table 2. All seven SNPs conformed to Hardy-Weinberg equilibrium in the control subjects (*P* > 0.05). Through the allelic model analysis, five SNPs were identified as closely related to steroid-induced ONFH by using the Pearson Chi-squared test. Allele T of rs99365, allele A of rs308952, allele C of rs3817004, allele C of rs2279750, and allele C of rs3755724 were, respectively, associated with a 0.73, 0.75, 0.76, 0.72, and 0.78-fold decreased steroid-induced ONFH risk (OR = 0.73, 95% CI [0.559–0.954], *p* = 0.02; OR = 0.75, 95% CI [0.571–0.37], *p* = 0.03; OR = 0.76, 95% CI [0.582–0.988], *p* = 0.04; OR = 0.72, 95% CI [0.55–0.936], *p* = 0.01; OR = 0.78, 95% CI [0.621–0.985], *p* = 0.04, respectively).

**Table 2:** Allele frequencies in cases and controls and odds ratio estimates for steroid-induced ONFH. The statistics of the allelic distributions, minor allele frequency (MAF), odds ratios (ORs), 95% confdence intervals (95% CIs) and the *P*-values of alleles are presented in Table 2.

			**Alleles**	**MAF**		***p*value for HWE**					
**SNP-ID**	**Gene**	**Position**	**A**^**a**^**/B**	**Case**	**Control**	**Case**	**Control**	**ORs**	**95% CI**		***p***
rs99365	TIMP4	12152585	T/C	0.216	0.274	0.488	1	0.73	0.559	0.954	**0.02^a^**
rs17035945	TIMP4	12153128	T/C	0.139	0.155	0.803	0.514	0.87	0.634	1.207	0.42
rs308952	TIMP4	12154122	A/G	0.219	0.274	1	1	0.75	0.571	0.973	**0.03^a^**
rs3817004	TIMP4	12154174	G/A	0.222	0.273	0.607	1	0.76	0.582	0.988	**0.04^a^**
rs28897670	TIMP4	12156160	G/A	0.094	0.115	0.726	1	0.80	0.553	1.167	0.25
rs2279750	TIMP4	12157117	C/A	0.218	0.279	1	0.888	0.72	0.55	0.936	**0.01^a^**
rs3755724	TIMP4	12159406	C/T	0.404	0.464	0.806	0.494	0.78	0.621	0.985	**0.04^a^**

**Notes.**

a Minor allele.

### The association of 7 SNPs genotypes in the TIMP4 genes with the gender and clinical phenotypes of ONFH

We completed the correlation analysis between the genotypes of the TIMP4 genes and gender, unilateral or bilateral hip lesions, and clinical stages of ONFH, respectively. Under the genotypes, rs3817004 showed a significant association with steroid-induced ONFH in gender, that is, in the heterozygous (GA) carriers of rs3817004 (G/A), the proportion (40.1%) of male was significantly increased compared with the proportion (30.1%) of female (*p* = 0.016). In the clinical phenotypes analysis of ONFH, the results revealed that all genotypes were not shown the statistical association, although the rs17035945 (T/C) with unilateral or bilateral hip lesions and clinical stages of ONFH also indicated statistical tendency (*p* = 0.602, *p* = 0.077, respectively) (Table 3).

**Table 3:** Stratified analysis of TIMP4 polymorphisms with the gender and clinical phenotypes of steroid-induced ONFH. Under the genotypes, rs3817004 showed a significant association with steroid-induced ONFH in gender, that is, in the heterozygous (GA) carriers of rs3817004 (G/A), the proportion (40.1%) of male was significantly increased compared with the proportion (30.1%) of female (*p* = 0.016). Bold values are statistically different (*p* ≤ 0.05).

**SNP-ID**		**Gender *n* (%)^a^**		**Hip lesions*n* (%)^a^**		**Clinical stages*n* (%)**^a^		
	**Genotype**	**Male**	**Female**	**Unilateral**	**Bilateral**	**II**	**III**	**IV**
rs99365	TT	4(2.3)	7(6,2)	1(1.2)	10(5)	2(3.2)	4(3.2)	5(5.2)
	TC	63(36.6)	38(33.6)	25(30.1)	76(37.6)	16(25.4)	55(43.7)	30(31.2)
	CC	105(61)	68(60.2)	57(68.7)	116(57.4)	45(71.4)	67(53.2)	61(63.5)
	*P*	0.243		0.117		0.097		
rs17035945	TT	3(1.7)	3(2.7)	1(1.2)	5(2.5)	0(0)	2(1.6)	4(4.2)
	TC	41(23.8)	26(23)	17(20.7)	50(24.6)	19(29.7)	22(17.5)	26(27.4)
	CC	128(74.4)	84(74.3)	64(78)	148(72.9)	45(70.3)	102(81)	65(68.4)
	*P*	0.866		0.602		0.077		
rs308952	AA	4(2.3)	9(8)	1(1.2)	12(5.9)	2(3.1)	5(4)	6(6.2)
	AG	64(37)	35(3.1)	25(30.1)	74(36.6)	17(26.6)	53(42.4)	29(30.2)
	GG	105(60.7)	68(60.7)	57(68.7)	116(57.4)	45(70.3)	67(53.6)	61(63.5)
	*P*	0.063		0.089		0.134		
rs3817004	GG	3(1.7)	9(8)	1(1.2)	11(5.4)	2(3.1)	5(4)	5(5.2)
	GA	69(40.1)	34(30.1)	26(31.3)	77(37.9)	18(28.1)	54(42.9)	31(32.3)
	AA	101(58.7)	70(61.9)	56(67.5)	115(56.7)	44(68.8)	67(53.2)	60(62.5)
	*P*	**0.016**		0.113		0.245		
rs28897670	GG	1(0.6)	2(1.8)	0(0)	3(1.5)	0(0)	1(0.8)	2(2.1)
	GA	31(17.9)	17(15)	13(15.7)	35(17.2)	14(21.9)	14(11.1)	20(20.8)
	AA	141(81.5)	94(83.2)	70(84.3)	165(81.3)	50(78.1)	111(88.1)	74(77.1)
	*P*	0.526		0.501		0.134		
rs2279750	CC	5(2.9)	8(7.1)	1(1.2)	12(5.9)	3(4.8)	6(4.8)	4(4.2)
	CA	62(36)	36(31.9)	26(31.3)	72(35.6)	16(25.4)	52(41.3)	30(31.2)
	AA	105(61)	69(61.1)	56(67.5)	118(58.4)	44(69.8)	68(54)	62(64.6)
	*P*	0.229		0.135		0.234		
rs3755724	CC	27(15.6)	21(18.6)	11(13.3)	37(18.2)	11(17.2)	20(15.9)	17(17.7)
	CT	88(50.9)	47(41.6)	36(43.4)	99(48.8)	26(40.6)	64(50.8)	45(46.9)
	TT	58(33.5)	45(39.8)	36(43.4)	67(33)	27(42.2)	42(33.3)	34(35.4)
	*P*	0.307		0.224		0.738		

**Notes.**

a X^2^ text.

### Risk assessment between the TIMP4 genotypic model and steroid-induced ONFH

For genotypic model analysis, five different genetic models were used to more deeply analyze the associations between the SNPs and the risk of steroid-induced ONFH. rs99365 showed significant association with decreased risk of steroid-induced ONFH in the Log-additive model (OR = 0.73, 95% CI [0.55–0.95], *p* = 0.02; adjusted OR = 0.74, 95% CI [0.56–0.99], *p* = 0.038). SNPs rs308952 and rs3817004 showed significant association with decreased risk of steroid-induced ONFH in the Log-additive model (OR = 0.74, 95% CI [0.57–0.97], *p* = 0.03, adjusted OR = 0.75, 95% CI [0.56–0.99], *p* = 0.044; OR = 0.75, 95% CI [0.58–0.99], *p* = 0.038, adjusted OR = 0.75, 95% CI [0.56–0.99], *p* = 0.042, respectively). rs2279750 showed significant association with decreased risk of steroid-induced ONFH in the Dominant model (OR = 0.68, 95% CI [0.49–0.94], *p* = 0.021; adjusted OR = 0.70, 95% CI [0.50–0.99], *p* = 0.042) and Log-additive model (OR = 0.71, 95% CI [0.55–0.93], *p* = 0.014; adjusted OR = 0.73, 95% CI [0.55–0.97], *p* = 0.028). Although crude analysis of rs3755724 showed decreased risk of steroid-induced ONFH in the Dominant model (OR = 0.68, 95% CI [0.48–0.96], *p* = 0.029) and Log-additive models (OR = 0.78, 95% CI [0.62–0.98], *p* = 0.035), there was no association after adjustment (Table 4).

**Table 4:** Genotypic model analysis of relationship between SNPs and steroid-induced ONFH risk. For genotypic model analysis, five different genetic models were used to more deeply analyze the associations between the SNPs and the risk of steroid-induced ONFH. Bold values are statistically different (*p* ≤ 0.05).

**SNP**	**Model**	**Genotype**	**Group=control**	**Group=case**	**Without adjustment**		**With adjustment**^a^	
					**OR(95% CI)**	***p***^b^**-value**	**OR(95% CI)**	***p***^b^**-value**
rs99365		C/C	162(52.8%)	173(60.7%)	1.00		1.00	
	Codominant	C/T	122(39.7%)	101(35.4%)	0.78(0.55–1.09)	0.054	0.78(0.55–1.11)	0.1
		T/T	23(7.5%)	11(3.9%)	0.45(0.21–0.95)		0.48(0.22–1.07)	
	Dominant	C/C	162(52.8%)	173(60.7%)	1.00	0.052	1.00	0.077
		C∕T − T∕T	145(47.2%)	112(39.3%)	0.72(0.52–1.00)		0.73(0.52–1.03)	
	Recessive	C∕C − C∕T	284(92.5%)	274(96.1%)	1.00	0.055	1.00	0.11
		T/T	23(7.5%)	11(3.9%)	0.50(0.24–1.04)		0.53(0.25–1.16)	
	Log-additive	—	—	—	**0.73(0.55–0.95)**	**0.02**	**0.74(0.56–0.99)**	**0.038**
rs308952		G/G	162(52.8%)	173(60.7%)	1.00		1.00	
	Codominant	A/G	122(39.7%)	99(34.7%)	0.76 (0.54–1.07)	0.093	0.77(0.54–1.09)	0.13
		A/A	23(7.5%)	13 (4.6%)	0.53 (0.26–1.08)		0.53(0.25–1.14)	
	Dominant	G/G	162 (52.8%)	173 (60.7%)	1.00	0.052	1.00	0.071
		A∕G − A∕A	145 (47.2%)	112 (39.3%)	0.72 (0.52–1.00)		0.73(0.52–1.03)	
	Recessive	G∕G − A∕G	284 (92.5%)	272 (95.4%)	1.00	0.13	1.00	0.16
		A/A	23 (7.5%)	13 (4.6%)	0.59 (0.29–1.19)		0.59(0.28–1.25)	
	Log-additive	—	—	—	**0.74(0.57–0.97)**	**0.03**	**0.75(0.56–0.99)**	**0.044**
rs3817004		A/A	163 (52.8%)	171 (59.8%)	1.00		1.00	
	Codominant	G/A	123 (39.8%)	103 (36%)	0.80(0.57–1.12)	0.1	0.79(0.55–1.13)	0.11
		G/G	23 (7.4%)	12 (4.2%)	0.50(0.24–1.03)		0.48(0.22–1.05)	
	Dominant	A/A	163 (52.8%)	171 (59.8%)	1.00	0.084	1.00	0.088
		G∕A − G∕G	146 (47.2%)	115 (40.2%)	0.75(0.54–1.04)		0.74(0.53–1.05)	
	Recessive	A∕A − G∕A	286 (92.6%)	274 (95.8%)	1.00	0.089	1.00	0.096
		G/G	23 (7.4%)	12 (4.2%)	0.54(0.27–1.12)		0.53(0.25–1.14)	
	Log-additive	—	—	—	**0.75(0.58–0.99)**	**0.038**	**0.75(0.56–0.99)**	**0.042**
rs2279750		A/A	159 (51.6%)	174 (61%)	1.00		1.00	
	Codominant	C/A	126 (40.9%)	98 (34.4%)	0.71(0.51–1.00)	0.047	0.73(0.51–1.05)	0.089
		C/C	23 (7.5%)	13 (4.6%)	0.52(0.25–1.05)		0.53(0.25–1.12)	
	Dominant	A/A	159 (51.6%)	174 (61%)	1.00	**0.021**	1.00	**0.042**
		C∕A − C∕C	149 (48.4%)	111 (39%)	**0.68 (0.49–0.94)**		**0.70(0.50–0.99)**	
	Recessive	A∕A − C∕A	285 (92.5%)	272 (95.4%)	1.00	0.14	1.00	0.16
		C/C	23 (7.5%)	13 (4.6%)	0.59(0.29–1.19)		0.59(0.28–1.25)	
	Log-additive	—	—	—	**0.71(0.55–0.93)**	**0.014**	**0.73(0.55–0.97)**	**0.028**
rs3755724		T/T	85 (27.7%)	103 (36%)	1.00		1.00	
	Codominant	T/C	159 (51.8%)	135 (47.2%)	0.70(0.49–1.01)	0.083	0.77(0.52–1.13)	0.29
		C/C	63 (20.5%)	48 (16.8%)	0.63(0.39–1.01)		0.71(0.43–1.17)	
	Dominant	T/T	85 (27.7%)	103 (36%)	1.00	**0.029**	1.00	0.12
		T∕C − C∕C	222 (72.3%)	183 (64%)	**0.68(0.48–0.96)**		0.75(0.52–1.08)	
	Recessive	T∕T − T∕C	244 (79.5%)	238 (83.2%)	1.00	0.24	1.00	0.43
		C/C	63 (20.5%)	48 (16.8%)	0.78(0.52–1.18)		0.84(0.54–1.30)	
	Log-additive	—	—	—	**0.78(0.62–0.98)**	**0.035**	0.83(0.65–1.06)	0.14

**Notes.**

a *p* value was adjusted by age.

### Risk assessment between the TIMP4 haplotype and steroid-induced ONFH

**Figure 1:**
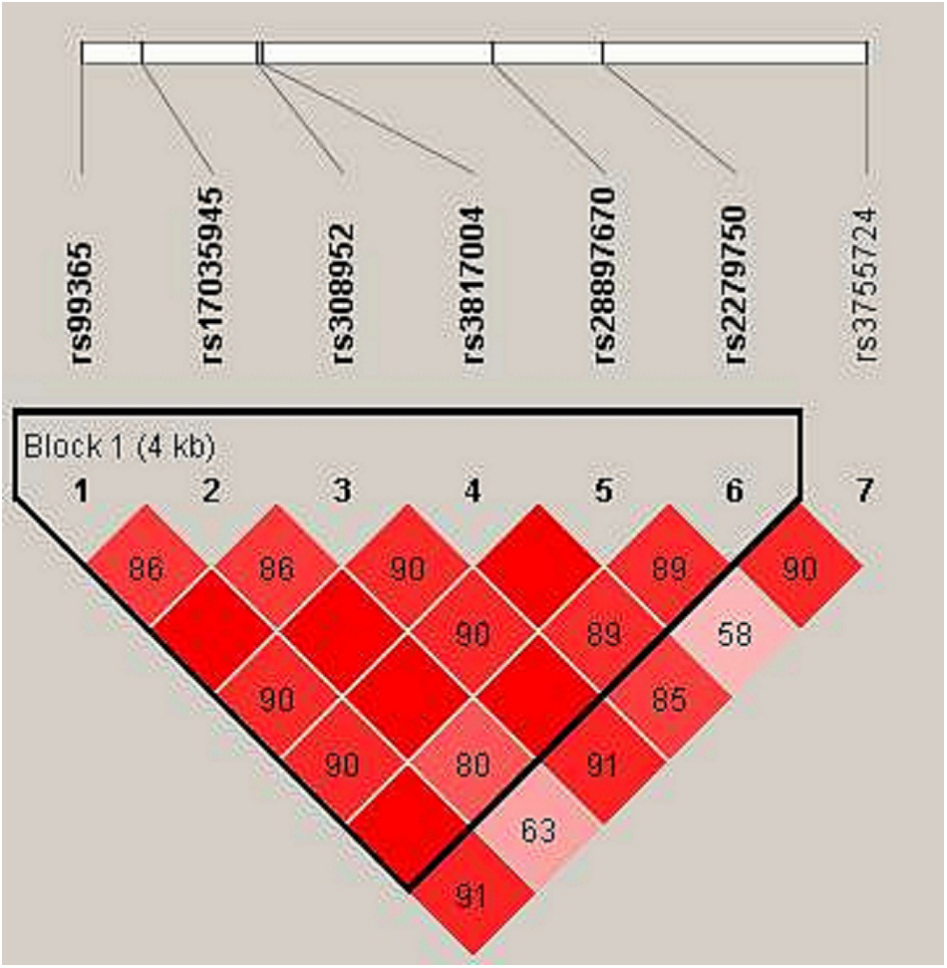
Linkage disequilibrium (LD) analysis of the SNPs on TIMP-4. Red squares display statistically significant associations between a pair of SNPs, as measured by D’; darker shades of red indicate higher D’.

In haplotype model analysis, one linkage disequilibrium (LD) block was detected in the TIMP4 SNPs (rs99365, rs17035945, rs308952, rs3817004, rs28897670 and rs227950; Fig. 1). Compared with the “CCGAAA” wild-type, the “TCAGAC” sequence was associated with a decreased risk of steroid-induced ONFH (OR = 0.71, 95% CI [0.53–0.95], *p* = 0.021; adjusted OR = 0.73, 95% CI [0.54–0.99], *p* = 0.04), and the “CCGGAA” sequence was also found to be associated with decreased risk after adjustment (OR = 0.31, 95% CI [0.10–0.98], *p* = 0.046) (Table 5).

**Table 5:** The haplotype frequencies of TIMP4 polymorphisms and their association with steroid-induced ONFH risk in case and control subjects. Compared with the “CCGAAA” wild-type, the “TCAGAC” sequence was associated with a decreased risk of steroid-induced ONFH (OR = 0.71, 95% CI [0.53–0.95], *p* = 0.021; adjusted OR = 0.73, 95% CI [0.54–0.99], *p* = 0.04), and the “CCGGAA” sequence was also found to be associated with decreased risk after adjustment (OR = 0.31, 95% CI [0.10–0.98], *P* = 0.046).

	**Haplotype**						**Freq**		**Without adjustment**		**With adjustment**	
	**rs99365**	**rs17035945**	**rs308952**	**rs3817004**	**rs28897670**	**rs227950**	**Case**	**Control**	**OR (95% CI)**	***p*-value**	**OR (95%CI)**	***p*-value**
1	C	C	G	A	A	A	0.625	0.557	1.00	—	1.00	—
2	T	C	A	G	A	C	0.208	0.254	**0.71(0.53–0.95)**	**0.021**	**0.73(0.54–0.99)**	**0.04**
3	C	T	G	A	G	A	0.090	0.105	0.72(0.48–1.09)	0.12	0.76(0.50–1.16)	0.21
4	C	T	G	A	A	A	0.051	0.039	1.12(0.64–1.96)	0.68	1.23(0.68–2.21)	0.49
5	C	C	G	G	A	A	0.009	0.020	0.34(0.11–1.05)	0.062	**0.31(0.10–0.98)**	**0.046**

## Discussion

In clinical orthopedic practice, ONFH is a refractory disease, and about 80% of untreated patients suffer from destructive femoral head collapse (Mont et al., 2006; Mont, Jones & Hungerford, 2006). Determining the molecular basis of pathogenesis has gradually become the focus of research on the prevention and treatment of ONFH. Human genetic polymorphisms affect the susceptibility and tolerance of the human body to disease, clinical phenotypic diversity, and response to drug treatment. There is a potential association between multiple genetic polymorphisms and susceptibility to ONFH, including polymorphisms in the PPAR *γ*, RUNX2, COL2A1, IGFBP3, MMP2, and MMP8 genes (Du et al., 2016; Song et al., 2017; Yu et al., 2016). As an endogenous inhibitor of MMPs, TIMP4 can effectively inhibit the expression of MMP-1, MMP-2, MMP-3, MMP-7, and MMP-9 (Liu et al., 1997), and gene variants may affect the risk of steroid induced ONFH. Through this case-control study, we explored the association between seven SNPs in TIMP4 and the risk of steroid-induced ONFH in the population of northern China . Based on our experimental results, SNPs rs99365, rs308952, rs3817004, rs2279750 and rs3755724 showed significant association with decreased susceptibility of steroid-induced ONFH. In addition, “TCAGAC” and “CCGGAA” sequences (rs99365, rs17035945, rs308952, rs3817004, rs28897670, and rs227950) were associated with 0.73-fold and 0.31-fold decreased steroid-induced ONFH risk respectively.

The appropriate assembly of the extracellular matrix (ECM) provides a suitable environment for the basic functions of cells, and changes of the ECM composition will affect embryonic development, morphogenesis, tissue remodeling, and repair (Brew & Nagase, 2010). There are four members of the TIMP family (TIMP1 to 4), which are involved in the degradation and composition of ECM (Bokarewa, Dahlberg & Tarkowski, 2005). The composition of ECM is dynamic, and this composition plays an important role in cartilage dynamic balance and cartilage repair (Ravindran & George, 2014; Yan & Zhang, 2006). The expression of TIMP4 also affects the damage and remodeling of cartilage and bone tissue. The expression of TIMP4 was markedly decreased in the cartilage of osteoarthritis patients (Kevorkian et al., 2004). However, according to an osteoarthritis study by Huang, TIMP-4 gene expression is increased in the cartilage of the human femoral head (Huang et al., 2002). In addition, Kumarasinghe et al. (2010) reported that that TIMP4 was significantly down-regulated in the bone of primary hip osteoarthritis, consistent with a role as a pivotal regulator of osteoblast expression. The necrosis of cartilage and subchondral bone eventually leads to the collapse of the femoral head, which is the main pathological change of ONFH (Song & Du, 2016). In ONFH, members of the MMPs/TIMPs family also participate in the accumulation and degradation of ECM in the cartilage and in the subchondral bone of the femoral head. In the extracellular matrix of cartilage, certain MMPs participate in the degradation of the ECM, and increased secretion of TIMP4 inhibits MMPs activity, to maintain cartilage self-renewal (Feng et al., 2013). Here, we report for the first time that five SNPs in TIMP4 are significantly associated with reduced risk of steroid-induced ONFH in the population of northern China. We speculate that TIMP4 likely involved in the pathological process of steroid-induced ONFH by regulating the extracellular matrix of the femoral head cartilage and subchondral bone.

Of the five SNPs in TIMP4 that showed significant association with decreased risk of steroid-induced ONFH, rs3755724 is located in the promoter region. Our results showed that allele C of rs3755724 was associated with a 0.78-fold decreased steroid-induced ONFH risk (OR = 0.78, 95% CI [0.621–0.985], *p* = 0.04). In the genotypic model analysis, rs3755724 was correlated with a decreased risk of steroid-induced ONFH in the Dominant model before adjustment. In a study of Korean children, an association of rs3755724 of TIMP4 with the development of Kawasaki disease with coronary artery lesions was identified (Ban et al., 2009). A study of cardiovascular disease showed that TIMP4 was up regulated in inflammatory processes of human cardiovascular pathology (Koskivirta et al., 2006). As an inhibitor of MMPs, TIMP4 is associated with smooth muscle cell migration and collagen deposition in vascular injury response (Dollery et al., 1999). Saito et al. studied the vasculitis caused by hormones and suggested that arteritis of the bone is a primary factor or a prerequisite for steroid-induced ONFH (Saito, Ohzono & Ono, 1992). TIMP4 is considered the main MMP inhibitor in human platelets, is involved in the regulation of platelet recruitment and aggregation, and its imbalance with MMP2 may be related to antithrombotic features (Radomski et al., 2002). In vulnerable subchondral capillaries and sinusoids, fibrin platelet thrombosis may be the main intermediate pathway of osteonecrosis of the femoral head (Jones Jr, 1993). Aller et al. (2017) reported the association of TIMP4 rs3755724 with predictors of ≥5% weight loss. Furthermore, in a study of obesity, TIMP4 was associated with high fat diet-induced obesity and may act through the regulation of lipid absorption (Sakamuri et al., 2017). There is a growing awareness that steroid hormones may cause ONFH through disruptions to lipid metabolism. Long-term high doses of steroid hormones intake induced hyperlipidemia and also caused hypertrophy and proliferation of fat cells in the bone marrow cavity of the femoral head, resulting in ONFH (Ding & Zhou, 2009). Therefore, we can speculate that TIMP4 may be involved in vasculitis formation, intravascular thrombosis and lipid metabolism disorders in hormone-mediated osteonecrosis of the femoral head.

## Conclusion

Our case-control study is the first demonstration that five TIMP4 SNPs (rs99365, rs308952, rs3817004, rs2279750, and rs3755724) are significantly associated with decreased risk of steroid-induced ONFH in the population of northern China. The identification of these alleles is an important step towards screening gene variants for the prediction of disease and early clinical prevention. However, the pathogenesis mechanisms of steroid-induced ONFH are very complex, and to clearly understand the exact mechanisms of steroid-induced ONFH and the function of TIMP4 in the associated pathological changes, more samples from different regions for more detail and in-depth studies are needed.

##  Supplemental Information

10.7717/peerj.6270/supp-1 Supplemental Information 1 The raw measurements The raw data indicate that five TIMP4 SNPs ( rs99365, rs308952, rs3817040, rs2279750 and rs3755724) are significantly associated with decreased risk of steroid-induced ONFH in the population of northern China.

## Additional Information and Declarations

### Data Availability

The following information was supplied regarding data availability: The raw data is available in a Supplemental File.
