# Supplementary material for: Association of TIMP4 gene variants with steroid-induced osteonecrosis of the femoral head in the population of northern China
Source: PeerJ. 2019 Jan 24;7:e6270. doi: 10.7717/peerj.6270 (PMC6348097; doi:10.7717/peerj.6270)
Supplement: Supplemental Information 1 — The raw data indicate that five TIMP4 SNPs ( rs99365, rs308952, rs3817040, rs2279750 and rs3755724) are significantly associated with decreased risk of steroid-induced ONFH in the population of northern China. [file peerj-07-6270-s001.zip › 5.docx]

CHR 3 BLOCK: rs99365、rs17035945、rs308952、rs3817004、rs28897670、rs2279750

| Haplotype | Freq. | Case, Control Ratio Counts | Case, Control Frequencies | Chi Square | P Value |
| --- | --- | --- | --- | --- | --- |
| CCGAAA | 0.59 | 353.8 : 212.2, 342.0 : 272.0 | 0.625, 0.557 | 5.653 | 0.0174 |
| TCAGAC | 0.232 | 117.9 : 448.1, 155.9 : 458.1 | 0.208, 0.254 | 3.434 | 0.0639 |
| CTGAGA | 0.098 | 50.9 : 515.1, 64.7 : 549.3 | 0.090, 0.105 | 0.806 | 0.3693 |
| CTGAAA | 0.045 | 29.1 : 536.9, 24.1 : 589.9 | 0.051, 0.039 | 1.025 | 0.3114 |
| CCGGAA | 0.015 | 5.1 : 560.9, 12.1 : 601.9 | 0.009, 0.020 | 2.356 | 0.1248 |

|  |
| --- |
| \| **Haplotype association with response (n=595, crude analysis)** \| \| \| \| \| \| \| \| \| \| \| \| \| --- \| --- \| --- \| --- \| --- \| --- \| --- \| --- \| --- \| --- \| --- \| --- \| \|  \| **rs99365** \| **rs17035945** \| **rs308952** \| **rs3817004** \| **rs28897670** \| **rs2279750** \| **Freq** \| **OR (95% CI)** \| **P-value** \|  \|  \| \| 1 \| C \| C \| G \| A \| A \| A \| 0.5878 \| 1.00 \| --- \|  \|  \| \| 2 \| T \| C \| A \| G \| A \| C \| 0.2325 \| **0.71 (0.53 - 0.95)** \| 0.021 \|  \|  \| \| 3 \| C \| T \| G \| A \| G \| A \| 0.0964 \| 0.72 (0.48 - 1.09) \| 0.12 \|  \|  \| \| 4 \| C \| T \| G \| A \| A \| A \| 0.0448 \| 1.12 (0.64 - 1.96) \| 0.68 \|  \|  \| \| 5 \| C \| C \| G \| G \| A \| A \| 0.0139 \| 0.34 (0.11 - 1.05) \| 0.062 \|  \|  \| \| rare \| * \| * \| * \| * \| * \| * \| 0.0244 \| 0.70 (0.29 - 1.70) \| 0.43 \|  \|  \| \| **Global haplotype association p-value:**0.069 \| \| \| \| \| \| \| \| \| \| \| \| |

| \|  \| \| --- \| |
| --- | --- |
| \| **Haplotype association with response (n=595, adjusted by Age+Gender)** \| \| \| \| \| \| \| \| \| \| \| \| \| --- \| --- \| --- \| --- \| --- \| --- \| --- \| --- \| --- \| --- \| --- \| --- \| \|  \| **rs99365** \| **rs17035945** \| **rs308952** \| **rs3817004** \| **rs28897670** \| **rs2279750** \| **Freq** \| **OR (95% CI)** \| **P-value** \|  \|  \| \| 1 \| C \| C \| G \| A \| A \| A \| 0.5874 \| 1.00 \| --- \|  \|  \| \| 2 \| T \| C \| A \| G \| A \| C \| 0.2326 \| **0.73 (0.54 - 0.99)** \| 0.04 \|  \|  \| \| 3 \| C \| T \| G \| A \| G \| A \| 0.0966 \| 0.76 (0.50 - 1.16) \| 0.21 \|  \|  \| \| 4 \| C \| T \| G \| A \| A \| A \| 0.045 \| 1.23 (0.68 - 2.21) \| 0.49 \|  \|  \| \| 5 \| C \| C \| G \| G \| A \| A \| 0.0144 \| **0.31 (0.10 - 0.98)** \| 0.046 \|  \|  \| \| rare \| * \| * \| * \| * \| * \| * \| 0.024 \| 0.73 (0.29 - 1.82) \| 0.5 \|  \|  \| \| **Global haplotype association p-value:**0.086 \| \| \| \| \| \| \| \| \| \| \| \| |
