# Supplementary material for: Association of TIMP4 gene variants with steroid-induced osteonecrosis of the femoral head in the population of northern China
Source: PeerJ. 2019 Jan 24;7:e6270. doi: 10.7717/peerj.6270 (PMC6348097; doi:10.7717/peerj.6270)
Supplement: Supplemental Information 1 — The raw data indicate that five TIMP4 SNPs ( rs99365, rs308952, rs3817040, rs2279750 and rs3755724) are significantly associated with decreased risk of steroid-induced ONFH in the population of northern China. [file peerj-07-6270-s001.zip › 1.docx]

Gender

| **Gender * Group Crosstabulation** | | | | | |
| --- | --- | --- | --- | --- | --- |
|  | | | Group | | Total |
|  |  |  | case | control |  |
| Gender | female | Count | 113 | 112 | 225 |
|  |  | % within Gender | 50.2% | 49.8% | 100.0% |
|  | male | Count | 173 | 197 | 370 |
|  |  | % within Gender | 46.8% | 53.2% | 100.0% |
| Total | | Count | 286 | 309 | 595 |
|  |  | % within Gender | 48.1% | 51.9% | 100.0% |

| **Chi-Square Tests** | | | | | |
| --- | --- | --- | --- | --- | --- |
|  | Value | df | Asymp. Sig. (2-sided) | Exact Sig. (2-sided) | Exact Sig. (1-sided) |
| Pearson Chi-Square | .673^a^ | 1 | .412 |  |  |
| Continuity Correction^b^ | .541 | 1 | .462 |  |  |
| Likelihood Ratio | .673 | 1 | .412 |  |  |
| Fisher's Exact Test |  |  |  | .447 | .231 |
| N of Valid Cases | 595 |  |  |  |  |

Age

| **Group Statistics** | | | | | |
| --- | --- | --- | --- | --- | --- |
|  | Group | N | Mean | Std. Deviation | Std. Error Mean |
| Age | control | 309 | 48.75 | 8.422 | .485 |
|  | case | 286 | 41.83 | 13.115 | .776 |

| **Independent Samples Test** | | | | | | | | | | |
| --- | --- | --- | --- | --- | --- | --- | --- | --- | --- | --- |
|  | | Levene's Test for Equality of Variances | | t-test for Equality of Means | | | | | | |
|  |  | F | Sig. | t | df | Sig. (2-tailed) | Mean Difference | Std. Error Difference | 95% Confidence Interval of the Difference | |
|  |  |  |  |  |  |  |  |  | Lower | Upper |
| Age | Equal variances assumed | 51.337 | .000 | 7.647 | 585 | .000 | 6.922 | .905 | 5.144 | 8.700 |
|  | Equal variances not assumed |  |  | 7.566 | 481.810 | .000 | 6.922 | .915 | 5.124 | 8.720 |
