# Supplementary material for: Association of TIMP4 gene variants with steroid-induced osteonecrosis of the femoral head in the population of northern China
Source: PeerJ. 2019 Jan 24;7:e6270. doi: 10.7717/peerj.6270 (PMC6348097; doi:10.7717/peerj.6270)
Supplement: Supplemental Information 1 — The raw data indicate that five TIMP4 SNPs ( rs99365, rs308952, rs3817040, rs2279750 and rs3755724) are significantly associated with decreased risk of steroid-induced ONFH in the population of northern China. [file peerj-07-6270-s001.zip › 3.docx]

| **Single-SNP analysis** |  |
| --- | --- |
| **SNP:**rs99365 |  |
| **Percentage of typed samples:**592/595 (99.5%)   \| **rs99365 association with response Group (n=592, crude analysis)** \| \| \| \| \| \| \| \| \| --- \| --- \| --- \| --- \| --- \| --- \| --- \| --- \| \| **Model** \| **Genotype** \| **Group=control** \| **Group=case** \| **OR (95% CI)** \| **P-value** \| **AIC** \| **BIC** \| \| Codominant \| C/C \| 162 (52.8%) \| 173 (60.7%) \| 1.00 \| 0.054 \| 820 \| 833.2 \| \| C/T \| 122 (39.7%) \| 101 (35.4%) \| 0.78 (0.55-1.09) \| \| T/T \| 23 (7.5%) \| 11 (3.9%) \| **0.45 (0.21-0.95)** \| \| Dominant \| C/C \| 162 (52.8%) \| 173 (60.7%) \| 1.00 \| 0.052 \| 820.1 \| 828.8 \| \| C/T-T/T \| 145 (47.2%) \| 112 (39.3%) \| 0.72 (0.52-1.00) \| \| Recessive \| C/C-C/T \| 284 (92.5%) \| 274 (96.1%) \| 1.00 \| 0.055 \| 820.2 \| 828.9 \| \| T/T \| 23 (7.5%) \| 11 (3.9%) \| 0.50 (0.24-1.04) \| \| Overdominant \| C/C-T/T \| 185 (60.3%) \| 184 (64.6%) \| 1.00 \| 0.28 \| 822.7 \| 831.5 \| \| C/T \| 122 (39.7%) \| 101 (35.4%) \| 0.83 (0.60-1.16) \| \| Log-additive \| --- \| --- \| --- \| **0.73 (0.55-0.95)** \| 0.02 \| 818.4 \| 827.2 \| |  |
| **SNP:**rs17035945 |  |
| **Percentage of typed samples:**594/595 (99.83%)   \| **rs17035945 association with response Group (n=594, crude analysis)** \| \| \| \| \| \| \| \| \| --- \| --- \| --- \| --- \| --- \| --- \| --- \| --- \| \| **Model** \| **Genotype** \| **Group=control** \| **Group=case** \| **OR (95% CI)** \| **P-value** \| **AIC** \| **BIC** \| \| Codominant \| C/C \| 222 (71.8%) \| 212 (74.4%) \| 1.00 \| 0.7 \| 827.8 \| 840.9 \| \| C/T \| 78 (25.2%) \| 67 (23.5%) \| 0.90 (0.62-1.31) \| \| T/T \| 9 (2.9%) \| 6 (2.1%) \| 0.70 (0.24-1.99) \| \| Dominant \| C/C \| 222 (71.8%) \| 212 (74.4%) \| 1.00 \| 0.49 \| 826 \| 834.8 \| \| C/T-T/T \| 87 (28.2%) \| 73 (25.6%) \| 0.88 (0.61-1.26) \| \| Recessive \| C/C-C/T \| 300 (97.1%) \| 279 (97.9%) \| 1.00 \| 0.53 \| 826.1 \| 834.9 \| \| T/T \| 9 (2.9%) \| 6 (2.1%) \| 0.72 (0.25-2.04) \| \| Overdominant \| C/C-T/T \| 231 (74.8%) \| 218 (76.5%) \| 1.00 \| 0.62 \| 826.2 \| 835 \| \| C/T \| 78 (25.2%) \| 67 (23.5%) \| 0.91 (0.63-1.32) \| \| Log-additive \| --- \| --- \| --- \| 0.88 (0.64-1.21) \| 0.42 \| 825.8 \| 834.6 \| |  |
| **SNP:**rs308952 |  |
| **Percentage of typed samples:**592/595 (99.5%)   \| **rs308952 association with response Group (n=592, crude analysis)** \| \| \| \| \| \| \| \| \| --- \| --- \| --- \| --- \| --- \| --- \| --- \| --- \| \| **Model** \| **Genotype** \| **Group=control** \| **Group=case** \| **OR (95% CI)** \| **P-value** \| **AIC** \| **BIC** \| \| Codominant \| G/G \| 162 (52.8%) \| 173 (60.7%) \| 1.00 \| 0.093 \| 821.1 \| 834.3 \| \| A/G \| 122 (39.7%) \| 99 (34.7%) \| 0.76 (0.54-1.07) \| \| A/A \| 23 (7.5%) \| 13 (4.6%) \| 0.53 (0.26-1.08) \| \| Dominant \| G/G \| 162 (52.8%) \| 173 (60.7%) \| 1.00 \| 0.052 \| 820.1 \| 828.8 \| \| A/G-A/A \| 145 (47.2%) \| 112 (39.3%) \| 0.72 (0.52-1.00) \| \| Recessive \| G/G-A/G \| 284 (92.5%) \| 272 (95.4%) \| 1.00 \| 0.13 \| 821.6 \| 830.4 \| \| A/A \| 23 (7.5%) \| 13 (4.6%) \| 0.59 (0.29-1.19) \| \| Overdominant \| G/G-A/A \| 185 (60.3%) \| 186 (65.3%) \| 1.00 \| 0.21 \| 822.3 \| 831.1 \| \| A/G \| 122 (39.7%) \| 99 (34.7%) \| 0.81 (0.58-1.13) \| \| Log-additive \| --- \| --- \| --- \| **0.74 (0.57-0.97)** \| 0.03 \| 819.1 \| 827.9 \| |  |
| **SNP:**rs3817004 |  |
| **Percentage of typed samples:**595/595 (100%)   \| **rs3817004 association with response Group (n=595, crude analysis)** \| \| \| \| \| \| \| \| \| --- \| --- \| --- \| --- \| --- \| --- \| --- \| --- \| \| **Model** \| **Genotype** \| **Group=control** \| **Group=case** \| **OR (95% CI)** \| **P-value** \| **AIC** \| **BIC** \| \| Codominant \| A/A \| 163 (52.8%) \| 171 (59.8%) \| 1.00 \| 0.1 \| 825.4 \| 838.5 \| \| G/A \| 123 (39.8%) \| 103 (36%) \| 0.80 (0.57-1.12) \| \| G/G \| 23 (7.4%) \| 12 (4.2%) \| 0.50 (0.24-1.03) \| \| Dominant \| A/A \| 163 (52.8%) \| 171 (59.8%) \| 1.00 \| 0.084 \| 825 \| 833.7 \| \| G/A-G/G \| 146 (47.2%) \| 115 (40.2%) \| 0.75 (0.54-1.04) \| \| Recessive \| A/A-G/A \| 286 (92.6%) \| 274 (95.8%) \| 1.00 \| 0.089 \| 825.1 \| 833.8 \| \| G/G \| 23 (7.4%) \| 12 (4.2%) \| 0.54 (0.27-1.12) \| \| Overdominant \| A/A-G/G \| 186 (60.2%) \| 183 (64%) \| 1.00 \| 0.34 \| 827 \| 835.8 \| \| G/A \| 123 (39.8%) \| 103 (36%) \| 0.85 (0.61-1.19) \| \| Log-additive \| --- \| --- \| --- \| **0.75 (0.58-0.99)** \| 0.038 \| 823.7 \| 832.4 \| |  |
| **SNP:**rs28897670 |  |
| **Percentage of typed samples:**595/595 (100%)   \| **rs28897670 association with response Group (n=595, crude analysis)** \| \| \| \| \| \| \| \| \| --- \| --- \| --- \| --- \| --- \| --- \| --- \| --- \| \| **Model** \| **Genotype** \| **Group=control** \| **Group=case** \| **OR (95% CI)** \| **P-value** \| **AIC** \| **BIC** \| \| Codominant \| A/A \| 242 (78.3%) \| 235 (82.2%) \| 1.00 \| 0.5 \| 828.6 \| 841.7 \| \| G/A \| 63 (20.4%) \| 48 (16.8%) \| 0.78 (0.52-1.19) \| \| G/G \| 4 (1.3%) \| 3 (1.1%) \| 0.77 (0.17-3.49) \| \| Dominant \| A/A \| 242 (78.3%) \| 235 (82.2%) \| 1.00 \| 0.24 \| 826.6 \| 835.3 \| \| G/A-G/G \| 67 (21.7%) \| 51 (17.8%) \| 0.78 (0.52-1.18) \| \| Recessive \| A/A-G/A \| 305 (98.7%) \| 283 (99%) \| 1.00 \| 0.78 \| 827.9 \| 836.7 \| \| G/G \| 4 (1.3%) \| 3 (1.1%) \| 0.81 (0.18-3.64) \| \| Overdominant \| A/A-G/G \| 246 (79.6%) \| 238 (83.2%) \| 1.00 \| 0.26 \| 826.7 \| 835.5 \| \| G/A \| 63 (20.4%) \| 48 (16.8%) \| 0.79 (0.52-1.19) \| \| Log-additive \| --- \| --- \| --- \| 0.80 (0.55-1.17) \| 0.25 \| 826.6 \| 835.4 \| |  |
| **SNP:**rs2279750 |  |
| **Percentage of typed samples:**593/595 (99.66%)   \| **rs2279750 association with response Group (n=593, crude analysis)** \| \| \| \| \| \| \| \| \| --- \| --- \| --- \| --- \| --- \| --- \| --- \| --- \| \| **Model** \| **Genotype** \| **Group=control** \| **Group=case** \| **OR (95% CI)** \| **P-value** \| **AIC** \| **BIC** \| \| Codominant \| A/A \| 159 (51.6%) \| 174 (61%) \| 1.00 \| 0.047 \| 821.1 \| 834.2 \| \| C/A \| 126 (40.9%) \| 98 (34.4%) \| 0.71 (0.51-1.00) \| \| C/C \| 23 (7.5%) \| 13 (4.6%) \| 0.52 (0.25-1.05) \| \| Dominant \| A/A \| 159 (51.6%) \| 174 (61%) \| 1.00 \| 0.021 \| 819.8 \| 828.6 \| \| C/A-C/C \| 149 (48.4%) \| 111 (39%) \| **0.68 (0.49-0.94)** \| \| Recessive \| A/A-C/A \| 285 (92.5%) \| 272 (95.4%) \| 1.00 \| 0.14 \| 823 \| 831.7 \| \| C/C \| 23 (7.5%) \| 13 (4.6%) \| 0.59 (0.29-1.19) \| \| Overdominant \| A/A-C/C \| 182 (59.1%) \| 187 (65.6%) \| 1.00 \| 0.1 \| 822.5 \| 831.3 \| \| C/A \| 126 (40.9%) \| 98 (34.4%) \| 0.76 (0.54-1.06) \| \| Log-additive \| --- \| --- \| --- \| **0.71 (0.55-0.93)** \| 0.014 \| 819.1 \| 827.8 \| |  |
| **SNP:**rs3755724 |  |
| **Percentage of typed samples:**593/595 (99.66%)   \| **rs3755724 association with response Group (n=593, crude analysis)** \| \| \| \| \| \| \| \| \| --- \| --- \| --- \| --- \| --- \| --- \| --- \| --- \| \| **Model** \| **Genotype** \| **Group=control** \| **Group=case** \| **OR (95% CI)** \| **P-value** \| **AIC** \| **BIC** \| \| Codominant \| T/T \| 85 (27.7%) \| 103 (36%) \| 1.00 \| 0.083 \| 822.4 \| 835.5 \| \| T/C \| 159 (51.8%) \| 135 (47.2%) \| 0.70 (0.49-1.01) \| \| C/C \| 63 (20.5%) \| 48 (16.8%) \| 0.63 (0.39-1.01) \| \| Dominant \| T/T \| 85 (27.7%) \| 103 (36%) \| 1.00 \| 0.029 \| 820.6 \| 829.4 \| \| T/C-C/C \| 222 (72.3%) \| 183 (64%) \| **0.68 (0.48-0.96)** \| \| Recessive \| T/T-T/C \| 244 (79.5%) \| 238 (83.2%) \| 1.00 \| 0.24 \| 824 \| 832.7 \| \| C/C \| 63 (20.5%) \| 48 (16.8%) \| 0.78 (0.52-1.18) \| \| Overdominant \| T/T-C/C \| 148 (48.2%) \| 151 (52.8%) \| 1.00 \| 0.26 \| 824.1 \| 832.9 \| \| T/C \| 159 (51.8%) \| 135 (47.2%) \| 0.83 (0.60-1.15) \| \| Log-additive \| --- \| --- \| --- \| **0.78 (0.62-0.98)** \| 0.035 \| 820.9 \| 829.7 \| |  |
