# Supplementary material for: Association of TIMP4 gene variants with steroid-induced osteonecrosis of the femoral head in the population of northern China
Source: PeerJ. 2019 Jan 24;7:e6270. doi: 10.7717/peerj.6270 (PMC6348097; doi:10.7717/peerj.6270)
Supplement: Supplemental Information 1 — The raw data indicate that five TIMP4 SNPs ( rs99365, rs308952, rs3817040, rs2279750 and rs3755724) are significantly associated with decreased risk of steroid-induced ONFH in the population of northern China. [file peerj-07-6270-s001.zip › 4.docx]

| **Single-SNP analysis** |  |
| --- | --- |
| **SNP:**rs99365 |  |
| **Percentage of typed samples:**592/595 (99.5%)   \| **rs99365 association with response Group (n=592, adjusted by Age+Gender)** \| \| \| \| \| \| \| \| \| --- \| --- \| --- \| --- \| --- \| --- \| --- \| --- \| \| **Model** \| **Genotype** \| **Group=control** \| **Group=case** \| **OR (95% CI)** \| **P-value** \| **AIC** \| **BIC** \| \| Codominant \| C/C \| 162 (52.8%) \| 173 (60.7%) \| 1.00 \| 0.1 \| 768.7 \| 790.6 \| \| C/T \| 122 (39.7%) \| 101 (35.4%) \| 0.78 (0.55-1.11) \| \| T/T \| 23 (7.5%) \| 11 (3.9%) \| 0.48 (0.22-1.07) \| \| Dominant \| C/C \| 162 (52.8%) \| 173 (60.7%) \| 1.00 \| 0.077 \| 768.1 \| 785.6 \| \| C/T-T/T \| 145 (47.2%) \| 112 (39.3%) \| 0.73 (0.52-1.03) \| \| Recessive \| C/C-C/T \| 284 (92.5%) \| 274 (96.1%) \| 1.00 \| 0.11 \| 768.6 \| 786.1 \| \| T/T \| 23 (7.5%) \| 11 (3.9%) \| 0.53 (0.25-1.16) \| \| Overdominant \| C/C-T/T \| 185 (60.3%) \| 184 (64.6%) \| 1.00 \| 0.3 \| 770.1 \| 787.6 \| \| C/T \| 122 (39.7%) \| 101 (35.4%) \| 0.83 (0.58-1.18) \| \| Log-additive \| --- \| --- \| --- \| **0.74 (0.56-0.99)** \| 0.038 \| 766.9 \| 784.4 \| |  |
| **SNP:**rs17035945 |  |
| **Percentage of typed samples:**594/595 (99.83%)   \| **rs17035945 association with response Group (n=594, adjusted by Age+Gender)** \| \| \| \| \| \| \| \| \| --- \| --- \| --- \| --- \| --- \| --- \| --- \| --- \| \| **Model** \| **Genotype** \| **Group=control** \| **Group=case** \| **OR (95% CI)** \| **P-value** \| **AIC** \| **BIC** \| \| Codominant \| C/C \| 222 (71.8%) \| 212 (74.4%) \| 1.00 \| 0.87 \| 772.9 \| 794.9 \| \| C/T \| 78 (25.2%) \| 67 (23.5%) \| 0.92 (0.62-1.37) \| \| T/T \| 9 (2.9%) \| 6 (2.1%) \| 0.83 (0.28-2.47) \| \| Dominant \| C/C \| 222 (71.8%) \| 212 (74.4%) \| 1.00 \| 0.62 \| 771 \| 788.5 \| \| C/T-T/T \| 87 (28.2%) \| 73 (25.6%) \| 0.91 (0.62-1.33) \| \| Recessive \| C/C-C/T \| 300 (97.1%) \| 279 (97.9%) \| 1.00 \| 0.77 \| 771.1 \| 788.7 \| \| T/T \| 9 (2.9%) \| 6 (2.1%) \| 0.85 (0.29-2.51) \| \| Overdominant \| C/C-T/T \| 231 (74.8%) \| 218 (76.5%) \| 1.00 \| 0.69 \| 771 \| 788.6 \| \| C/T \| 78 (25.2%) \| 67 (23.5%) \| 0.92 (0.62-1.37) \| \| Log-additive \| --- \| --- \| --- \| 0.92 (0.66-1.28) \| 0.6 \| 770.9 \| 788.5 \| |  |
| **SNP:**rs308952 |  |
| **Percentage of typed samples:**592/595 (99.5%)   \| **rs308952 association with response Group (n=592, adjusted by Age+Gender)** \| \| \| \| \| \| \| \| \| --- \| --- \| --- \| --- \| --- \| --- \| --- \| --- \| \| **Model** \| **Genotype** \| **Group=control** \| **Group=case** \| **OR (95% CI)** \| **P-value** \| **AIC** \| **BIC** \| \| Codominant \| G/G \| 162 (52.8%) \| 173 (60.7%) \| 1.00 \| 0.13 \| 768.9 \| 790.8 \| \| A/G \| 122 (39.7%) \| 99 (34.7%) \| 0.77 (0.54-1.09) \| \| A/A \| 23 (7.5%) \| 13 (4.6%) \| 0.53 (0.25-1.14) \| \| Dominant \| G/G \| 162 (52.8%) \| 173 (60.7%) \| 1.00 \| 0.071 \| 767.7 \| 785.2 \| \| A/G-A/A \| 145 (47.2%) \| 112 (39.3%) \| 0.73 (0.52-1.03) \| \| Recessive \| G/G-A/G \| 284 (92.5%) \| 272 (95.4%) \| 1.00 \| 0.16 \| 769 \| 786.5 \| \| A/A \| 23 (7.5%) \| 13 (4.6%) \| 0.59 (0.28-1.25) \| \| Overdominant \| G/G-A/A \| 185 (60.3%) \| 186 (65.3%) \| 1.00 \| 0.24 \| 769.6 \| 787.1 \| \| A/G \| 122 (39.7%) \| 99 (34.7%) \| 0.81 (0.57-1.15) \| \| Log-additive \| --- \| --- \| --- \| **0.75 (0.56-0.99)** \| 0.044 \| 766.9 \| 784.4 \| |  |
| **SNP:**rs3817004 |  |
| **Percentage of typed samples:**595/595 (100%)   \| **rs3817004 association with response Group (n=595, adjusted by Age+Gender)** \| \| \| \| \| \| \| \| \| --- \| --- \| --- \| --- \| --- \| --- \| --- \| --- \| \| **Model** \| **Genotype** \| **Group=control** \| **Group=case** \| **OR (95% CI)** \| **P-value** \| **AIC** \| **BIC** \| \| Codominant \| A/A \| 163 (52.8%) \| 171 (59.8%) \| 1.00 \| 0.11 \| 771.3 \| 793.3 \| \| G/A \| 123 (39.8%) \| 103 (36%) \| 0.79 (0.55-1.13) \| \| G/G \| 23 (7.4%) \| 12 (4.2%) \| 0.48 (0.22-1.05) \| \| Dominant \| A/A \| 163 (52.8%) \| 171 (59.8%) \| 1.00 \| 0.088 \| 770.9 \| 788.4 \| \| G/A-G/G \| 146 (47.2%) \| 115 (40.2%) \| 0.74 (0.53-1.05) \| \| Recessive \| A/A-G/A \| 286 (92.6%) \| 274 (95.8%) \| 1.00 \| 0.096 \| 771 \| 788.6 \| \| G/G \| 23 (7.4%) \| 12 (4.2%) \| 0.53 (0.25-1.14) \| \| Overdominant \| A/A-G/G \| 186 (60.2%) \| 183 (64%) \| 1.00 \| 0.34 \| 772.8 \| 790.4 \| \| G/A \| 123 (39.8%) \| 103 (36%) \| 0.84 (0.59-1.20) \| \| Log-additive \| --- \| --- \| --- \| **0.75 (0.56-0.99)** \| 0.042 \| 769.6 \| 787.2 \| |  |
| **SNP:**rs28897670 |  |
| **Percentage of typed samples:**595/595 (100%)   \| **rs28897670 association with response Group (n=595, adjusted by Age+Gender)** \| \| \| \| \| \| \| \| \| --- \| --- \| --- \| --- \| --- \| --- \| --- \| --- \| \| **Model** \| **Genotype** \| **Group=control** \| **Group=case** \| **OR (95% CI)** \| **P-value** \| **AIC** \| **BIC** \| \| Codominant \| A/A \| 242 (78.3%) \| 235 (82.2%) \| 1.00 \| 0.52 \| 774.5 \| 796.4 \| \| G/A \| 63 (20.4%) \| 48 (16.8%) \| 0.78 (0.50-1.20) \| \| G/G \| 4 (1.3%) \| 3 (1.1%) \| 0.94 (0.20-4.46) \| \| Dominant \| A/A \| 242 (78.3%) \| 235 (82.2%) \| 1.00 \| 0.27 \| 772.5 \| 790.1 \| \| G/A-G/G \| 67 (21.7%) \| 51 (17.8%) \| 0.79 (0.51-1.20) \| \| Recessive \| A/A-G/A \| 305 (98.7%) \| 283 (99%) \| 1.00 \| 0.99 \| 773.8 \| 791.3 \| \| G/G \| 4 (1.3%) \| 3 (1.1%) \| 0.99 (0.21-4.66) \| \| Overdominant \| A/A-G/G \| 246 (79.6%) \| 238 (83.2%) \| 1.00 \| 0.26 \| 772.5 \| 790 \| \| G/A \| 63 (20.4%) \| 48 (16.8%) \| 0.78 (0.50-1.20) \| \| Log-additive \| --- \| --- \| --- \| 0.82 (0.55-1.21) \| 0.31 \| 772.7 \| 790.3 \| |  |
| **SNP:**rs2279750 |  |
| **Percentage of typed samples:**593/595 (99.66%)   \| **rs2279750 association with response Group (n=593, adjusted by Age+Gender)** \| \| \| \| \| \| \| \| \| --- \| --- \| --- \| --- \| --- \| --- \| --- \| --- \| \| **Model** \| **Genotype** \| **Group=control** \| **Group=case** \| **OR (95% CI)** \| **P-value** \| **AIC** \| **BIC** \| \| Codominant \| A/A \| 159 (51.6%) \| 174 (61%) \| 1.00 \| 0.089 \| 769.7 \| 791.6 \| \| C/A \| 126 (40.9%) \| 98 (34.4%) \| 0.73 (0.51-1.05) \| \| C/C \| 23 (7.5%) \| 13 (4.6%) \| 0.53 (0.25-1.12) \| \| Dominant \| A/A \| 159 (51.6%) \| 174 (61%) \| 1.00 \| 0.042 \| 768.4 \| 785.9 \| \| C/A-C/C \| 149 (48.4%) \| 111 (39%) \| **0.70 (0.50-0.99)** \| \| Recessive \| A/A-C/A \| 285 (92.5%) \| 272 (95.4%) \| 1.00 \| 0.16 \| 770.6 \| 788.1 \| \| C/C \| 23 (7.5%) \| 13 (4.6%) \| 0.59 (0.28-1.25) \| \| Overdominant \| A/A-C/C \| 182 (59.1%) \| 187 (65.6%) \| 1.00 \| 0.16 \| 770.6 \| 788.1 \| \| C/A \| 126 (40.9%) \| 98 (34.4%) \| 0.78 (0.55-1.11) \| \| Log-additive \| --- \| --- \| --- \| **0.73 (0.55-0.97)** \| 0.028 \| 767.7 \| 785.2 \| |  |
| **SNP:**rs3755724 |  |
| **Percentage of typed samples:**593/595 (99.66%)   \| **rs3755724 association with response Group (n=593, adjusted by Age+Gender)** \| \| \| \| \| \| \| \| \| --- \| --- \| --- \| --- \| --- \| --- \| --- \| --- \| \| **Model** \| **Genotype** \| **Group=control** \| **Group=case** \| **OR (95% CI)** \| **P-value** \| **AIC** \| **BIC** \| \| Codominant \| T/T \| 85 (27.7%) \| 103 (36%) \| 1.00 \| 0.29 \| 771.2 \| 793.1 \| \| T/C \| 159 (51.8%) \| 135 (47.2%) \| 0.77 (0.52-1.13) \| \| C/C \| 63 (20.5%) \| 48 (16.8%) \| 0.71 (0.43-1.17) \| \| Dominant \| T/T \| 85 (27.7%) \| 103 (36%) \| 1.00 \| 0.12 \| 769.3 \| 786.8 \| \| T/C-C/C \| 222 (72.3%) \| 183 (64%) \| 0.75 (0.52-1.08) \| \| Recessive \| T/T-T/C \| 244 (79.5%) \| 238 (83.2%) \| 1.00 \| 0.43 \| 771 \| 788.6 \| \| C/C \| 63 (20.5%) \| 48 (16.8%) \| 0.84 (0.54-1.30) \| \| Overdominant \| T/T-C/C \| 148 (48.2%) \| 151 (52.8%) \| 1.00 \| 0.41 \| 771 \| 788.5 \| \| T/C \| 159 (51.8%) \| 135 (47.2%) \| 0.87 (0.62-1.22) \| \| Log-additive \| --- \| --- \| --- \| 0.83 (0.65-1.06) \| 0.14 \| 769.5 \| 787.1 \| |  |
